# Different determinants of exercise capacity in HFpEF compared to HFrEF

**DOI:** 10.1186/s12947-017-0103-x

**Published:** 2017-04-26

**Authors:** Arlind Batalli, Pranvera Ibrahimi, Ibadete Bytyçi, Artan Ahmeti, Edmond Haliti, Shpend Elezi, Michael Y. Henein, Gani Bajraktari

**Affiliations:** 1 Clinic of Cardiology, University Clinical Centre of Kosova, “Rrethi i Spitalit”, p.n., Prishtina, Kosovo; 2 Medical Faculty, University of Prishtina, Prishtina, Kosovo; 3 Department of Public Health and Clinical Medicine, Umeå University and Heart Centre, Umeå, Sweden; 4 Molecular and Clinical Sciences Research Institute, St George University London, London, United Kingdom

**Keywords:** Six-minute walk test, Doppler echocardiography, Heart failure, HFpEF, Exercise capacity

## Abstract

**Background:**

Quality of life is as important as survival in heart failure (HF) patients. Controversies exist with regards to echocardiographic determinants of exercise capacity in HF, particularly in patients with preserved ejection fraction (HFpEF). The aim of this study was to prospectively examine echocardiographic parameters that correlate and predict functional exercise capacity assessed by 6 min walk test (6-MWT) in patients with HFpEF.

**Methods:**

In 111 HF patients (mean age 63 ± 10 years, 47% female), an echo-Doppler study and a 6-MWT were performed in the same day. Patients were divided into two groups based on the 6-MWT distance (Group I: ≤ 300 m and Group II: >300 m).

**Results:**

Group I were older (*p* = 0.008), had higher prevalence of diabetes (*p* = 0.027), higher baseline heart rate (*p* = 0.004), larger left atrium - LA (*p* = 0.001), longer LV filling time - FT (*p* = 0.019), shorter isovolumic relaxation time (*p* = 0.037), shorter pulmonary artery acceleration time - PA acceleration time (*p* = 0.006), lower left atrial lateral wall myocardial velocity (a’) (*p* = 0.018) and lower septal systolic myocardial velocity (s’) (*p* = 0.023), compared with Group II.

Patients with HF and reduced EF (HFrEF) had lower hemoglobin (*p* = 0.007), higher baseline heart rate (*p* = 0.005), higher NT-ProBNP (*p* = 0.001), larger LA (*p* = 0.004), lower septal s’, e’, a’ waves, and septal mitral annular plane systolic excursion (MAPSE), shorter PA acceleration time (*p <* 0.001 for all), lower lateral MAPSE, higher E/A & E/e’, and shorter LVFT (*p* = 0.001 for all), lower lateral e’ (*p* = 0.009), s’ (*p* = 0.006), right ventricular e’ and LA emptying fraction (*p* = 0.012 for both), compared with HFpEF patients.

In multivariate analysis, only LA diameter [2.676 (1.242–5.766), *p* = 0.012], and diabetes [0.274 (0.084–0.898), *p* = 0.033] independently predicted poor 6-MWT performance in the group as a whole. In HFrEF, age [1.073 (1.012–1.137), *p* = 0.018] and LA diameter [3.685 (1.348–10.071), *p* = 0.011], but in HFpEF, lateral s’ [0.295 (0.099–0.882), *p* = 0.029], and hemoglobin level [0.497 (0.248–0.998), *p* = 0.049] independently predicted poor 6-MWT performance.

**Conclusions:**

In HF patients determinants of exercise capacity differ according to severity of overall LV systolic function, with left atrial enlargement in HFrEF and longitudinal systolic shortening in HFpEF as the the main determinants.

## Background

Despite advances in the diagnosis and treatment of heart failure (HF), it still presents a major public health problem [1], with increased incidence [2, 3] and poor prognosis [4–6]. In patients with HF and reduced left ventricular (LV) ejection fraction (HFrEF) several echo parameters correlated with functional capacity [7–16]. In contrast, in patients with HF and preserved LV EF (HFpEF), determinants of functional capacity are not well investigated [15–18]. Exercise capacity has been objectively assessed in the setting of HF using the six-min walk test (6-MWT) [19]. While several echocardiographic markers at rest predicted limited exercise capacity in patients with HFrEF [7–16], none of them correlate with functional capacity in those with HFpEF [15, 17]. The aim of this study was to prospectively examine whether 6-MWT results correlate with cardiac function parameters in a consecutive group of patients with HF and to identify possible determinants of exercise capacity in those with HFpEF.

## Methods

### Study population

We studied 111 patients (mean age 63 ± 10 years, 47% female), with clinical diagnosis of HF, and New York Heart Association (NYHA) functional class I-III, secondary to ischemic or non-ischemic etiology. Patients were referred to the Clinic of Cardiology, University Clinical Centre of Kosova, between May 2013 and June 2016. At the time of the study all patients were on optimum HF medications, optimized at least 2 weeks prior to enrollment, based on patient’s symptoms and renal function: 82% were receiving ACE inhibitors or ARB, 78% beta-blockers, 12% calcium-blockers, 10% digoxin, 52% spironolactone, 62% diuretics. Patients with HFrEF had ischemic aetiology in 45%, hypertensive in 38%, and unknown aetiology in 17%. Patients with HFpEF had ischemic aetiology in 41% and hypertensive in 59%. All patients were in sinus rhythm. Patients with clinical evidence for cardiac decompensation, limited physical activity due to factors other than cardiac symptoms (e.g. arthritis), more than moderate mitral regurgitation, more than mild renal failure, chronic obstructive pulmonary disease or those with recent acute coronary syndrome, stroke or anemia were excluded. Patients gave a written informed consent to participate in the study, which was approved by the local Ethics Committee.

### Data collection

Detailed history and clinical assessment were obtained in all patients, in whom routine biochemical tests were also performed including hemoglobin, lipid profile, blood glucose level, and kidney function tests. Estimated body mass index (BMI) was calculated from weight and height measurements. Waist, hip measurements were also made and waist/hip ratio was calculated.

### Echocardiographic examination

A single operator performed all echocardiographic examinations using a Philips Intelligent E-33 system with a multi-frequency transducer, and harmonic imaging as appropriate. Images were obtained with the patient in the left lateral decubitus position and during quiet expiration. Measurements of interventricular septal thickness, posterior wall thickness, and LV dimensions were made at end-diastole and end-systole, as recommended by the American Society of Echocardiography [19]. LV mass (LVM) was calculated using the Devereux formula [20] and normalized to body surface area (LV mass index [LVMI]).

LV volumes and EF were calculated from the apical 2 and 4 chamber views using the modified Simpson’s method. Ventricular long axis motion was studied by placing the M-mode cursor at the lateral and septal angles of the mitral ring and the lateral angle of the tricuspid ring. Total amplitude of long axis motion was measured as previously described [21] from peak inward to peak outward points. LV and right ventricular (RV) long axis myocardial velocities were also studied using Doppler myocardial imaging technique. From the apical 4-chamber view, longitudinal velocities were recorded with the sample volume placed at the basal part of LV lateral and septal segments as well as RV free wall. Systolic (s’), as well as early and late (e’ and a’) diastolic myocardial velocities were measured with the gain optimally adjusted. Mean value of lateral and septal LV velocities were calculated.

Left atrial diameter was measured from aortic root recordings with the M-mode cursor positioned at the level of the aortic valve leaflets. LA volumes were measured using area-length method from the apical four chamber views, according to the guidelines of the American Society of Echocardiography and European Association of Echocardiography [22]. Left atrial maximal volume (LAV max) was measured at the end of LV systole, just before the opening of the mitral valve, LA minimal volume (LAV min) was measured at end diastole, right after mitral valve closure. LA emptying fraction (LA EF) was calculated with the formula [22, 23]:

$$ L A\  total\  emptying\  fraction = L A V\  max\ \hbox{--}\ L A V\  min/ L A V\  max \times 100 $$

Diastolic LV and RV function was assessed from filling velocities using spectral pulsed wave Doppler with the sample volume positioned at the tips of the mitral and tricuspid valve leaflets, respectively, during a brief apnea. Peak LV and RV early (E wave) and late (A wave) diastolic velocities were measured and E/A ratios were calculated. E wave deceleration time (DT) was also measured from peak E wave to the end of its deceleration in all study patients. The E/e’ ratio was calculated from the transmitral E wave and the mean lateral and septal segments e’ wave velocities. The isovolumic relaxation time was also measured from aortic valve closure to mitral valve opening, on the pulsed wave Doppler recording. LV filling pattern was considered ‘restrictive’ when E/A ratio was >2.0, E wave deceleration time < 140 ms and the left atrium dilated of more than 40 mm in transverse diameter [24]. Total LV filling time was measured from the onset of the E wave to the end of the A wave and ejection time from the onset to the end of the aortic Doppler flow velocity.

Mitral regurgitation severity was assessed by colour and continuous wave Doppler and was graded as mild, moderate, or severe according to the relative jet area to that of the left atrium as well as the flow velocity profile, in line with the recommendations of the American and European Society of Echocardiography [25, 26]. Likewise, tricuspid regurgitation was assessed by colour Doppler and continuous-wave Doppler. Retrograde trans-tricuspid pressure drop > 35 mmHg was taken as an evidence for pulmonary hypertension [26, 27]. All M-mode and Doppler recordings were made at a fast speed of 100 mm/s with a superimposed ECG (lead II).

### Measurement of amino-terminal pro BNP

Fasting venous blood was collected from study participants after they had rested in a supine position for 20 min. Samples were placed in disposable EDTA containers (1 g/L of plasma), and N-terminal proBNP was measured by a Cobas Elecsys E 411 analyzer (measuring range 5–35000 pg/mL) using a chemiluminescent immunoassay kit (Roche Diagnostics, Grenach-Wyhlen, Germany).

### Six minute walk test

Within 24 h of the echocardiographic examination a 6-MWT was performed on a level hallway surface, administered by a specialized nurse who was blinded to the results of the echocardiogram. According to the method of Gyatt et al. [28] patients were informed of the purpose and protocol of the 6 MWT which was conducted in a standardized fashion while patients on their regular medications [29, 30]. A 15 m flat, obstacle-free corridor was used and patients were instructed to walk as far as they can, turning 180° after they have reached the end of the corridor, during the allocated time of 6 min. Patients walked unaccompanied so not to influence walking speed. At the end of the 6 min the supervising nurse measured the total distance walked by the patient.

Using the norm-reference equation developed by Troosters [31] for the prediction of 6MWT distance according to age, height, weight, and gender that has been proposed for healthy patients, we derived the percentage of the predicted value (PPV). PPV is computed by dividing the actual 6MWT distance by the expected value of 6MWT distance and then multiplying by 100. Troosters’ equation is as follows: Predicted 6MWT distance = 218 + 5.14 ^∗^ height (cm) − 5.32 ^∗^ age (years) −1.8 ^∗^ weight (kg) + 51.31 ^∗^ sex (1–male, 0–female).

### Statistical analysis

Data are presented as mean ± SD or proportions (% of patients). Continuous data was compared with two-tailed unpaired Student’s *t* test and discrete data with Chi-square test. Correlations were tested with Pearson coefficients. Determinants of 6 MWT distance were identified with univariate analysis and multivariate logistic regression was performed using the step-wise method. A significant difference was defined as *p <* 0.05 (2-tailed). Patients were divided according to their ability to walk >300 m into good and limited exercise performance groups [30], and were compared using unpaired Student *t*-test. Also, patients with HFpEF (>45%) were compared with those with HFrEF (<45%) using unpaired *t*-test.

## Results

### Patients with Limited vs. Good 6 MWT performance (Table 1)

**Table 1:** Patients with limited exercise vs. good exercise capacity (6-MWT distance)

Variable	All patients	6MWT > 300 m	6MWT ≤ 300 m	*P* value
	(*n =* 111)	(*n =* 70)	(*n =* 41)	
Clinical and biochemical data				
Age (years)	63 ± 10	61 ± 10	66 ± 9	0.008
Female (%)	47	41	56	0.135
Smoking (%)	31.5	30	34	0.676
Diabetes (%)	28	20	41	0.027
Arterial hypertension (%)	69.4	68.6	70.7	0.835
Waist/hips ratio	0.97 ± 0.06	0.96 ± 0.06	0.98 ± 0.05	0.064
BMI (kg/m^2^)	28.6 ± 4.1	28.7 ± 4.2	28.4 ± 3.8	0.764
BSA (m^2^)	1.13 ± 0.2	1.15 ± 0.1	1.08 ± 0.1	0.067
Fasting glucose (mmol/L)	6.8 ± 2.8	6.4 ± 2.4	7.9 ± 2.9	0.009
Total cholesterol (mmol/L)	4.9 ± 1.2	4.9 ± 1.1	4.8 ± 1.2	0.439
Triglycerides (mmol/L)	1.7 ± 0.7	1.7 ± 0.7	1.6 ± 0.8	0.747
Creatinine (μmol/L)	96 ± 46	98 ± 56	93 ± 21	0.527
Hemoglobin (g/dl)	12.7 ± 1.8	12.8 ± 1.6	12.3 ± 2.0	0.166
NT-ProBNP (pg/mL	1178 ± 1635	970 ± 1286	1534 ± 2072	0.124
Baseline heart rate (beats/min)	74 ± 16	71 ± 15	80 ± 13	0.004
Echocardiographic data				
Ejection fraction (%)	47 ± 15	48 ± 14	45 ± 15	0.409
IVSd (cm)	1.1 ± 0.2	1.1 ± 0.2	1.11 ± 0.1	0.923
Left atrium (cm)	4.4 ± 0.8	4.2 ± 0.7	4.8 ± 0.9	0.001
LV EDD (cm)	5.8 ± 1.0	5.7 ± 0.8	5.9 ± 1.1	0.319
LV ESD (cm)	4.4 ± 1.2	4.2 ± 1.1	4.5 ± 1.3	0.281
Lateral MAPSE (cm)	1.15 ± 0.4	1.2 ± 0.4	1.05 ± 0.3	0.051
Septal MAPSE (cm)	1.0 ± 0.3	1.0 ± 0.3	0.9 ± 0.2	0.054
TAPSE (cm)	2.15 ± 0.5	2.2 ± 0.4	2.1 ± 0.5	0.466
LVPWd (cm)	1.05 ± 0.15	1.05 ± 0.2	1.05 ± 0.1	0.960
LVM (g)	266 ± 85	260 ± 75	279 ± 101	0.324
LVMI (g/m^2.7^)	58 ± 21	53 ± 16	67 ± 26	0.006
E/A ratio	1.05 ± 0.7	1.0 ± 0.7	1.1 ± 0.8	0.414
E wave DT	174 ± 49	178 ± 50	167 ± 44	0.228
Filling time (ms)	414 ± 132	434 ± 141	376 ± 104	0.019
IVRT (ms)	126 ± 41	131 ± 42	110 ± 34	0.037
PA acceleration time (ms)	110 ± 24	114 ± 23	100 ± 21	0.006
E/e’ ratio	11 ± 5.9	10 ± 4.1	13 ± 8.0	0.053
Lateral e’ (cm/s)	6.1 ± 2.1	6.1 ± 2.4	6.0 ± 2.6	0.817
Lateral a’(cm/s)	7.9 ± 3.6	8.1 ± 3.7	7.5 ± 3.5	0.461
Lateral s’ (cm/s)	5.3 ± 1.6	5.5 ± 1.5	4.9 ± 1.6	0.101
Septal e’ (cm/s)	4.9 ± 2.2	5.1 ± 2.2	4.6 ± 2.1	0.312
Septal a’ (cm/s)	7.15 ± 2.3	7.5 ± 2.4	6.4 ± 1.8	0.018
Septal s’ (cm/s)	4.4 ± 1.5	4.6 ± 1.6	4.0 ± 1.0	0.023
Right e’ (cm/s)	8.7 ± 3.0	8.9 ± 3.1	8.3 ± 2.7	0.305
Right a’ (cm/s)	12.5 ± 4.4	12.7 ± 4.3	11.8 ± 4.3	0.333
Right s’ (cm/s)	8.8 ± 3.0	9.8 ± 2.8	8.0 ± 3.2	0.086
LAV max (ml)	73 ± 35	68 ± 26	80 ± 47	0.218
LAV min (ml)	40 ± 29	35 ± 20	49 ± 38	0.067
LA EF (%)	47 ± 18	49 ± 17	45 ± 16	0.314

*LV* left ventricle, *EDD* end-diastolic dimension, *ESD* end-systolic dimension, *DT* deceleration time, *FT* filling time, *ET* Ejection time, *HR* heart rate, *IVSd* interventricular septum in diastole, *LVPWd* left ventricular posterior wall in diastole, *MAPSE* mitral annular plane systolic excursion, *TAPSE* tricuspid annular plane systolic excursion, *PA* pulmonary artery, *A* atrial diastolic velocity, *E* early diastolic filling velocity, *e’* early diastolic myocardial velocity, *s’* systolic myocardial velocity, *LA* left atrium, *LAV max* left atrial maximal volume, *LAV min* left atrial minimal volume, *LA EF* left atrial emptying fraction, *LVM* left ventricular mass, *LVMI* left ventricular mass index

Patients with limited exercise capacity were older (*p* = 0.008) and had higher prevalence of diabetes (*p* = 0.027) compared with those with good exercise capacity. Patients with limited 6-MWT had larger left atrium (*p* = 0.001), increased LVMI (*p* = 0.006), shorter LV filling time (*p* = 0.019), shorter isovolumic relaxation time (*p* = 0.037) and shorter PA acceleration time (*p* = 0.006), lower septal a’ (*p* = 0.018) and s’ (*p* = 0.023), compared to those with good 6-MWT performance. The rest of the clinical and echocardiographic indices were not different between groups.

### Patients with HFpEF vs. HFrEF (Table 2)

**Table 2:** Comparison of patients’ data between patients HFpEF and HFrEF

Variable	HFpEF	HFrEF	*P* value
	(*n =* 55)	(*n =* 56)	
Clinical and biochemical data			
Age (years)	63 ± 6.8	62 ± 12	0.767
Body-mass index (kg/m^2^)	29 ± 4	28 ± 3.6	0.130
Waist/hips ratio	0.96 ± 0.06	0.96 ± 0.06	0.996
Fasting glucose (mmol/L)	7.1 ± 3.1	6.7 ± 2.3	0.462
Total cholesterol (mmol/L)	5.1 ± 1.1	4.7 ± 1.1	0.054
Triglycerides (mmol/L)	1.8 ± 0.7	1.5 ± 0.7	0.137
Creatinine (μmol/L)	87 ± 16	105 ± 63	0.052
Hemoglobin (g/dL)	12.8 ± 1.9	12.4 ± 1.5	0.078
Baseline heart rate (beats/min)	70 ± 15	79 ± 15	0.005
NT-ProBNP (pg/mL)	681 ± 1280	1665 ± 1801	0.001
6-min walk distance (m)	339 ± 106	282 ± 119	0.010
PPV of 6-min walk distance (%)	56 ± 19	45 ± 20	0.004
Echocardiographic data			
IVSd (cm)	1.1 ± 0.1	1.1 ± 0.2	0.666
Left atrium (cm)	4.2 ± 0.8	4.6 ± 0.7	0.004
LV EDD (cm)	5.2 ± 0.7	6.3 ± 0.9	<0.001
LV ESD (cm)	3.5 ± 0.7	5.1 ± 1	<0.001
EDV (ml)	135 ± 44	213 ± 67	<0.001
ESV (ml)	56 ± 8.7	140 ± 52	<0.001
LV EF (%)	59.6 ± 8.7	35 ± 7.5	<0.001
Lateral MAPSE (cm)	1.27 ± 0.3	1.02 ± 0.4	0.001
Septal MAPSE (cm)	1.14 ± 0.2	0.85 ± 0.2	<0.001
TAPSE (cm)	2.3 ± 0.4	2.1 ± 0.5	0.062
LVPWd (cm)	1.05 ± 0.2	1.06 ± 0.2	0.688
E/A ratio	0.81 ± 0.3	1.3 ± 0.9	0.001
LVM (g)	224 ± 69	310 ± 77	<0.001
LVMI (g/m^2.7^)	50 ± 16	67 ± 23	<0.001
E wave DT	189 ± 44	159 ± 49	0.002
Filling time (ms)	454 ± 147	372 ± 99	0.001
IVRT (ms)	122 ± 39	130 ± 43	0.407
PA acceleration time (ms)	118 ± 20	101 ± 23	<0.001
E/e’ ratio	9.4 ± 4.7	13.5 ± 6.4	0.001
Lateral e’ (cm/s)	6.7 ± 2.6	5.3 ± 2.2	0.009
Lateral a’ (cm/s)	8.6 ± 3.3	7.1 ± 3.8	0.051
Lateral s’ (cm/s)	5.7 ± 1.3	4.8 ± 1.7	0.006
Septal e’ (cm/s)	5.7 ± 2.5	4.0 ± 1.1	<0.001
Septal a’ (cm/s)	7.9 ± 2.1	6.2 ± 2.0	<0.001
Septal s’ (cm/s)	4.9 ± 1.6	3.8 ± 0.9	<0.001
Right e’ (cm/s)	9.4 ± 3.2	7.9 ± 2.4	0.012
Right a’ (cm/s)	12.7 ± 4.5	12.2 ± 4.2	0.593
Right s’ (cm/s)	9.1 ± 3.1	8.3 ± 2.7	0.187
*LAV max*	62 ± 23	83 ± 57	0.005
*LAV min*	29 ± 18	51 ± 34	<0.001
LA EF (%)	53 ± 15	43 ± 18	0.012

*LV* left ventricle, *EDD* end-diastolic dimension, *ESD* end-systolic dimension, *DT* deceleration time, *FT* filling time, *ET* Ejection time, *HR* heart rate, *IVSd* interventricular septum in diastole, *LVPWd* left ventricular posterior wall in diastole, *MAPSE* mitral annular plane systolic excursion, *TAPSE* tricuspid annular plane systolic excursion, *PA* pulmonary artery, *A* atrial diastolic velocity, *E* early diastolic filling velocity, *e’* early diastolic myocardial velocity, *s’* systolic myocardial velocity, *LA* left atrial, *LAV max* left atrial maximal volume, *LAV min* left atrial minimal volume, *LA EF* left atrial eemptying fraction, *LVM* left ventricular mass, *LVMI* left ventricular mass index, *PPV* percentage of the predicted value

**Fig. 1:**
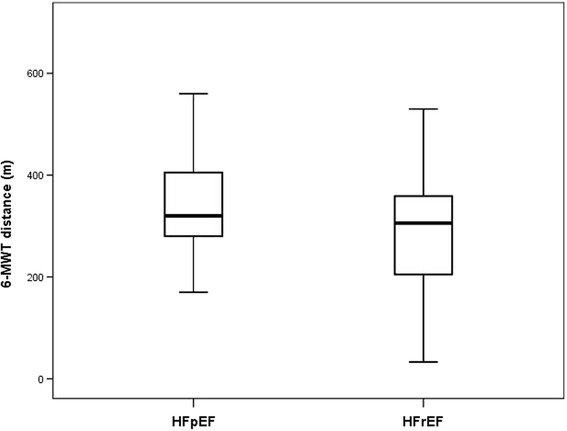
Six-minute walk test (6-MWT) distance in patients with heart failure and preserved ejection fraction (HFpEF) and in patients with heart failure and reduced ejection fraction (HFrEF)

The whole group of study patients walked a distance of 310 ± 116 m during 6-MWT. Patients with HFrEF walked significantly shorter distance compared those with HFpEF (*p* = 0.01, Fig. 1).

Mean PPV of 6-MWT distance for the whole group was 50 ± 20%, and was lower in HFrEF compared to HFpEF patients (*p* = 0.004). Patients with HFrEF had lower hemoglobin (*p* = 0.007), higher baseline heart rate (*p* = 0.005), higher NT-ProBNP (*p* = 0.001), larger LAV max (*p* = 0.005), larger LAV min (*p <* 0.001), larger LA (*p* = 0.004), increased LVM and LVMI (*p <* 0.001, for both), shorter pulmonary acceleration time, lower septal s’, e’ and a’ velocities, and lower septal and lateral mitral annular plane systolic excursion (MAPSE) (*p <* 0.001 for all), higher E/A, shorter LVFT and higher E/e’ (*p* = 0.001 for all), shorter E wave DT (*p* = 0.002), lower lateral e’ (*p* = 0.009) and s’ (*p* = 0.006), RV e’ and LA EF (*p* = 0.012 for both) compared to HFpEF patients. Eleven of the 55 HFpEF patients and 23 of 56 HFrEF patients had mild-moderate mitral regurgitation. Seven of the 55 HFpEF patients and 17 of 56 HFrEF patients had mild or more tricuspid regurgitation.

### Determinants of limited 6 MWT distance (Table 3)

**Table 3:** Determinants of limited exercise in HF patients

Variable	OR	(CI 95%)	*P* value
Determinants of all HF study patients			
Univariate determinants			
Age	1.062	(1.014–1.112)	0.011
Diabetes mellitus	0.353	(0.150–0.892)	0.017
NYHA class >1	0.290	(0.108–0.783)	0.015
LVMI	1.035	(1.011–1.060)	0.004
Left atrium	2.410	(1.404–4.137)	0.001
E wave	1.023	(1.004–1.043)	0.019
FT	0.996	(0.993–1.000)	0.036
Heart rate	1.039	(1.010–1.069)	0.008
PAAC	0.972	(0.952–0.993)	0.010
E/e’	1.092	(1.009–1.181)	0.028
Septal a’	0.786	(0.631–0.979)	0.032
Septal s’	0.661	(0.444–0.984)	0.041
Multivariate determinants			
Left atrium diameter	2.676	(1.242–5.766)	0.012
Diabetes mellitus	0.274	(0.084–0.898)	0.033
Age	1.067	(0.999–1.140)	0.052
NYHA class >1	2.068	(0.859–4.978)	0.105
Gender	0.406	(0.122–1.350)	0.141
E/e’	1.043	(0.943–1.153)	0.415
FT	0.997	(0.989–1.005)	0.463
Septal s’	0.854	(0.512–1.422)	0.543
Heart rate	1.011	(0.940–1.088)	0.764
Determinants in HFpEF patients			
Univariate determinants			
Diabetes mellitus	0.276	(0.082–0.926)	0.037
Haemoglobin	0.697	(0.502–0.968)	0.031
NYHA class >1	0.206	(0.043–0.993)	0.049
BSA	0.005	(0.000–0.308)	0.012
LVMI	1.049	(1.006–1.094)	0.025
Lateral a’	0.772	(0.603–0.987)	0.039
Lateral s’	0.489	(0.270–0.886)	0.018
Multivariate determinants			
Lateral s’	0.295	(0.099–0.882)	0.029
Haemoglobin	0.497	(0.248–0.998)	0.049
NYHA class >1	0.051	(0.003–1.034)	0.053
BSA	0.081	(0.000–6.016)	0.463
Lateral a’	1.049	(0.734–1.500)	0.793
Age	0.988	(0.827–1.179)	0.891
Diabetes	0.860	(0.109–6.786)	0.886
Determinants in HFrEF patients			
Univariate determinants			
Age	1.067	(1.010–1.127)	0.020
Left atrium	3.236	(1.333–7.856)	0.009
LAV max	1.021	(1.001–1.042)	0.045
LAV min	1.029	(1.003–1.055)	0.032
Multivariate determinants			
Age	1.073	(1.012–1.137)	0.018
Left atrium diameter	3.685	(1.348–10.071)	0.011
Gender	2.147	(0.556–8.288)	0.268

*BMI* body mass index, *BSA* body surface area, *NYHA* New York Heart Association, *LV* left ventricle, *EDD* end-diastolic dimension, *ESD* end-systolic dimension, *FT* filling time, *ET* ejection time, *PA* pulmonary artery, *A* atrial diastolic velocity, *E* early diastolic filling velocity, *e’* early diastolic myocardial velocity, *s’* systolic myocardial velocity, *LVMI* left ventricular mass index

#### Determinants of limited 6 MWT distance in all HF patients

High baseline heart rate (*p* = 0.008), age (*p* = 0.011), diabetes (*p* = 0.017) and NYHA class (*p* = 0.015) predicted limited 6-MWT distance. Also, enlarged LA (*p* = 0.001), increased LVMI (*p* = 0.004), increased E wave velocity (*p* = 0.019), short LV filling time (*p* = 0.036) and pulmonary artery acceleration time (*p* = 0.01), raised E/e’ (*p* = 0.028), low septal a’ and s’ (*p* = 0.032 and *p* = 0.041, respectively), predicted limited 6 MWT distance. In multivariate analysis [odds ratio 95% confidence interval], only enlarged LA diameter [2.676 (1.242–5.766), *p* = 0.012], and diabetes [0.274 (0.084–0.898), *p* = 0.033], independently predicted the limited 6-MWT distance.

#### Determinants of limited 6 MWT distance in HFpEF patients

**Fig. 2:**
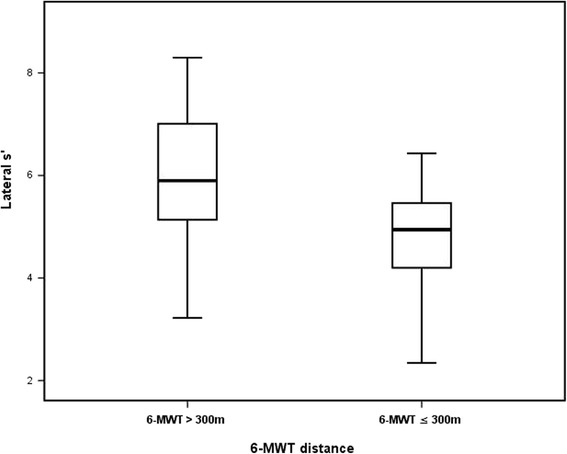
Systolic myocardial velocity (s’) of the lateral left ventricular wall in patients with limited vs. good performance exercise capacity in patients with heart failure and preserved left ventricular ejection fraction

In univariate analysis, body surface area - BSA (*p* = 0.012), low hemoglobin level (*p* = 0.031), diabetes (*p* = 0.037), and NYHA class > 1 (*p* = 0.049), increased LVMI (*p* = 0.025), low lateral s’ (*p* = 0.018) and a’ (*p* = 0.039) predicted limited 6-MWT distance. In multivariate analysis, lateral s’ [0.295 (0.099–0.882), *p* = 0.029, Fig. 2], and hemoglobin level [0.497 (0.248–0.998), *p* = 0.049], independently predicted the limited 6-MWT distance.

#### Determinants of limited 6 MWT distance in HFrEF patients

**Fig. 3:**
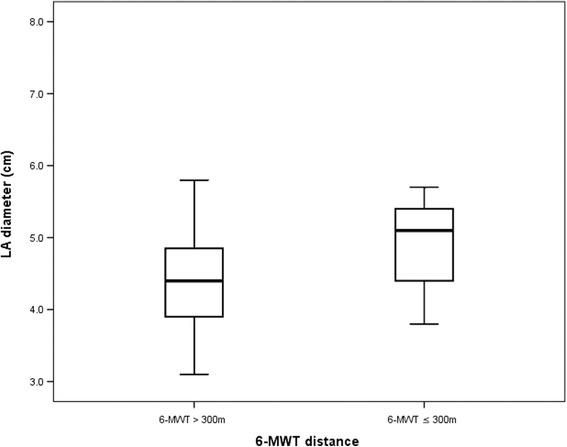
Left atrium diameter in patients with good vs. limited performance exercise capacity in patients with heart failure and reduced left ventricular ejection fraction

In univariate analysis, age (*p* = 0.02) and enlarged LA (*p* = 0.009) predicted limited exercise distance, which also remained as independent determinants in multivariate analysis: age [1.073 (1.012–1.137), *p* = 0.018] and LA diameter [3.685 (1.348–10.071), *p* = 0.011, Fig. 3].

## Discussion

### Findings

The results of this study show that in general HF patients with limited exercise capacity are older and have worse left ventricular function and raised filling pressures than those with satisfactory exercise performance. However, determinants of exercise capacity differed significantly according to ejection fraction. While patients with reduced ejection fraction have the enlarged left atrium and advanced age as the independent determinants of exercise capacity, it was the low hemoglobin and reduced lateral s’ the respective determinants in those with preserved ejection fraction.

### Data interpretation

Patients with heart failure due to reduced ejection fraction are known to have worse segmental and overall ventricular function, with additional signs of myocardial stiffness and raised filling pressures in many of them [32]. These perpetual changes result in left atrial enlargement due to the raised pressure, either because of venous hypertension, additional mitral regurgitation or the combination of both [33]. Indeed left atrial enlargement has previously been shown to be the most important prognostic marker in heart failure patients, irrespective of the development of atrial fibrillation [34]. It has also been taken as a reflection of the severity of LV myocardial stiffness, which is an end-stage dysfunction, thus an irreversible damage. On the other hand, many factors contribute to the pathophysiology of heart failure with preserved ejection fraction [35], including atrial fibrillation, hypertension and kidney disease. Although none of our patients was in atrial fibrillation, the low hemoglobin levels were the main determinant of compromised exercise capacity. This reflects the need for acknowledging differences in the strategic management of these patients when compared with those of HFrEF. Finally, our findings discard ejection fraction, as the commonest marker of ventricular function as a determinant of exercise capacity.

#### Limitations

The main limitation of our study is that we did not investigate the response of echocardiographic measurements to exercise, at the time of symptoms development. However the objective of this study was to determine determinants of ordinary walking exercise limitation rather than heavy exercise in HF patients. The other limitation was the lack of invasive measurements of left atrial pressures, but the study was based on Doppler measurements which have been shown to be reproducible and correlate closely with invasive pressure measurements [36]. The small sample size was another limitation, but we believe that future studies with larger sample size should strength our findings.

#### Clinical implications

Patients with HF have significantly limited exercise tolerance. Although ejection fraction is considered as the most useful index of LV function and the corner stone for recruiting patients for various treatment modalities, the other echo parameters should be considered as part of the conventional protocol of the follow-up of such patients, depending on overall LV systolic function: enlarged left atrium in HFrEF and impaired longitudinal systolic shortening and reduced hemoglobin in those with HFpEF. While management of patients with HFrEF could be standardized, and follow one protocol, that of patients with HFpEF is likely to be individualized.

## Conclusions

In HF patients determinants of exercise capacity differ according to severity of overall LV systolic function, with left atrial enlargement in HFrEF and longitudinal systolic shortening and low hemoglobin in HFpEF as the the main determinants.

## Abbreviations

6-MWT: Minute walk test
A: Atrial diastolic velovity
a’: Atrial myocardial velocity
BMI: Body mass index
BSA: Body surface area
DT: Deceleration time
e’: Early diastolic myocardial velocity
E: Early diastolic velocity
EF: Ejection fraction
FT: Filling time
HF: Heart failure
HFpEF: Heart failure with preserved ejection fraction
HFrEF: Heart failure with reduced ejection fraction
LA EF: Left atrial emptying fraction
LA: Left atrium
LAV max: Left atrial maximal volume
LAV min: Left atrial minimal volume
LV: Left ventricle
LVM: Left ventricular mass
LVMI: Left ventricular mass index
LVPWd: Left ventricular posterior wall in diastole
MAPSE: Mitral annular plane systolic excursion
NYHA: New York Heart Association
PA: Pulmonary artery
PPV: Percentage of the predicted value
RV: Right ventricle
s’: Systolic myocardial velocity
TAPSE: Tricuspidal annular plane systolic excursion
